# *In Vivo* Fluorescence-Based Endoscopic Detection of Colon Dysplasia in the Mouse Using a Novel Peptide Probe

**DOI:** 10.1371/journal.pone.0017384

**Published:** 2011-03-08

**Authors:** Sharon J. Miller, Bishnu P. Joshi, Ying Feng, Adam Gaustad, Eric R. Fearon, Thomas D. Wang

**Affiliations:** 1 Department of Internal Medicine, University of Michigan, Ann Arbor, Michigan, United States of America; 2 Department of Biomedical Engineering, University of Michigan, Ann Arbor, Michigan, United States of America; 3 Department of Human Genetics, University of Michigan, Ann Arbor, Michigan, United States of America; 4 Department of Pathology, University of Michigan, Ann Arbor, Michigan, United States of America; 5 Cancer Center, University of Michigan, Ann Arbor, Michigan, United States of America; University of Texas Southwestern Medical Center at Dallas, United States of America

## Abstract

Colorectal cancer (CRC) is a major cause of cancer-related deaths in much of the world. Most CRCs arise from pre-malignant (dysplastic) lesions, such as adenomatous polyps, and current endoscopic screening approaches with white light do not detect all dysplastic lesions. Thus, new strategies to identify such lesions, including non-polypoid lesions, are needed. We aim to identify and validate novel peptides that specifically target dysplastic colonic epithelium *in vivo*. We used phage display to identify a novel peptide that binds to dysplastic colonic mucosa *in vivo* in a genetically engineered mouse model of colo-rectal tumorigenesis, based on somatic *Apc* (*adenomatous polyposis coli*) gene inactivation. Binding was confirmed using confocal microscopy on biopsied adenomas and excised adenomas incubated with peptide *ex vivo*. Studies of mice where a mutant *Kras* allele was somatically activated in the colon to generate hyperplastic epithelium were also performed for comparison. Several rounds of *in vivo* T7 library biopanning isolated a peptide, QPIHPNNM. The fluorescent-labeled peptide bound to dysplastic lesions on endoscopic analysis. Quantitative assessment revealed the fluorescent-labeled peptide (target/background: 2.17±0.61) binds ∼2-fold greater to the colonic adenomas when compared to the control peptide (target/background: 1.14±0.15), p<0.01. The peptide did not bind to the non-dysplastic (hyperplastic) epithelium of the *Kras* mice. This work is first to image fluorescence-labeled peptide binding *in vivo* that is specific towards colonic dysplasia on wide-area surveillance. This finding highlights an innovative strategy for targeted detection to localize pre-malignant lesions that can be generalized to the epithelium of hollow organs.

## Introduction

Colorectal cancer (CRC) is the second leading cause of cancer death in the U.S. The average lifetime risk of CRC is 1 in 20 within the industrialized world, with the highest rate of incidence being within the U.S. (approximately 142,000 new cases diagnosed in 2010) [1], [2]. Adenomatous polyps, or adenomas, appear to be major precursors to CRC, though only a fraction of adenomas progress to CRC. The current screening method for CRC and adenomas uses standard white light endoscopy to detect morphological changes and lesions in the mucosa. Average polyp miss rates have been reported as high as 22%, with flat and depressed lesions being the most difficult to identify with conventional white light colonoscopy [3]–[7]. Furthermore, the presence of flat dysplastic lesions in the setting of chronic ulcerative colitis presents a significantly increased risk for the development of frank carcinoma [8]. These statistics support the fact that the morbidity rate from CRC could be significantly reduced with new targeted methods of early cancer detection based on protein expression rather than on anatomical changes.

Pre-clinical mouse models of disease provide an important tool for studying mechanisms of disease development. It has been established that mutations in the *adenomatous polyposis coli* (*APC*) gene are likely to be critical events in the initiation of the majority of adenomas and CRC [9]–[11]. Previously reported genetically engineered mouse models that mimic human *APC* gene mutations mainly develop adenomas in the small intestine (e.g. *Apc^Min^* model), rather than the distal colon, making it difficult to image the progression of polyps *in vivo* using small animal endoscopy. In the mouse model used in this study (*CPC;Apc* model [12]), one *Apc* allele is somatically inactivated in the epithelium from the distal ileum to the rectum. As described previously, the mice develop between 4 to 10 adenomas in the distal colon and rectum. Of note, the adenomas present within these mice not only develop from somatic modification of a gene that underlies adenoma and carcinoma development, but a subset also progresses to carcinoma, akin to the situation in man.

While adenomas have been shown as precursors of CRC, hyperplastic polyps have not. The progression of an adenoma to CRC can be attributed to accumulated genetic defects that regulate homeostatic cell behavior. Mutant *KRAS* alleles have been shown to promote tumorigenic growth in CRC cells, but do not in cases of hyperplasia where the malignant potential is negligible [13]. In this study, we also used another mouse model having an activated *Kras^G12D^* mutant allele that demonstrates hyperplastic polyp-like features, to investigate fluorescent-labeled peptide binding to a hyperplastic model that does not progress to carcinoma.

Peptides that bind to pre-cancerous colorectal lesions have the potential to guide tissue biopsy [14], and such peptides can be isolated using combinatorial phage display screening [14], [15]. Although much effort has concentrated on small-molecule and antibody ligands, peptides have advantages for *in vivo* use in the gastrointestinal tract, because they can be delivered topically to identify early molecular changes in the epithelium, where adenomas and carcinomas originate. In addition, peptides have minimal immunogenicity and can exhibit rapid binding kinetics and diffuse into diseased mucosa. Phage display is a powerful combinatorial technique for peptide discovery that uses methods of recombinant DNA technology to generate a complex library of peptides, often expressing up to 10^7^–10^9^ unique sequences, that can bind to cell surface targets [16], [17]. The DNA of candidate phages can be recovered and sequenced, elucidating positive binding peptides that can then be synthetically fabricated in large quantities at relatively low cost. The T7 system has proven effective for *in vivo* panning experiments identifying peptides specific to pancreatic islet vasculature [18], breast vasculature [19], bladder tumor cells [20], and hepatocytes [21]. Panning with intact tissue presents additional relevant cell targets while accounting for subtle features of the tissue microenvironment that may affect binding [15], [22]–[24].

Our aims here are to select peptides that preferentially bind to adenomas in the *CPC;Apc* mouse model using *in vivo* phage display and to demonstrate this binding *in vivo* using a fluorescence-label on small animal endoscopy. After peptide selection, we aim to show that the fluorescence-labeled peptide binds preferentially to dysplastic epithelium in the *CPC;Apc* mice and not to non-dysplastic epithelium from control mice or to hyperplastic colonic epithelium seen following somatic activation of a mutant *Kras* allele in colonic epithelium.

## Results

### *In vivo* phage display panning isolated QPIHPNNM as a binder to colonic dysplasia

**Figure 1:**
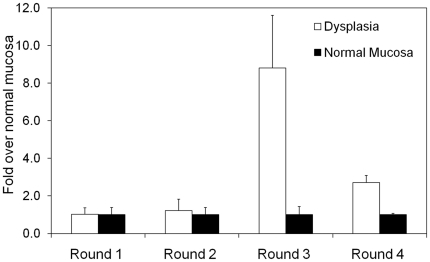
Titer results showing plaque forming units (pfu) for adenomas normalized to the number of phages bound to normal colonic mucosa. After two rounds of panning, the recovered phage pool was first cleared against a homogenized mixture of organs (heart, kidney, liver, normal colon) and subsequently cleared against normal colon tissue only. Clearing decreased the number of phage that bound to normal colon tissue 9× or 3× in rounds 3 and 4, respectively. The titer was performed in triplicate.

After the first two rounds of *in vivo* panning, the number of phages bound to the colonic adenomas was approximately the same as that for the adjacent normal colon tissue (Fig. 1). However, after clearing against normal colon and other organs, the third round showed a 9-fold increase in the number of bound phages to the adenomas over adjacent normal colon tissue. This trend, though not as pronounced, continued after the fourth round of panning. Fifty phages were selected from rounds three and four of panning, and the DNA was sequenced. Three entire sequences were repeated twice (NGTTSSNNQLINENNIQN, EHMYNTPHTYHTTMKNNK, QPIHPNNM) and four partial sequences were repeated twice (NKLAAALE, KNYKN, TNTHN, KHTNN). The QPIHPNNM peptide was only found in round 3, whereas the two full sequences and the four partial sequences were found in both rounds 3 and 4. An advantage of the T7 phage library system is that it does not have phage sequence amplification bias as compared to other phage libraries [25], thus any repeated phages were considered promising candidates. One of the three repeated phages was less than 18 amino acids because of the presence of a stop codon within the sequence. Using the RELIC software, the INFO value for the three candidate phages were calculated against a pool of phages from the initial T7 library [26]. The INFO scores for the NGTTSSNNQLINENNIQN, EHMYNTPHTYHTTMKNNK, and QPIHPNNM were 41.6, 45.2, and 49.1, respectively. A higher INFO score suggests a greater probability that the phage clone identified was not by chance. From this data, the QPIHPNNM peptide was the most unique sequence of the three longer sequences recovered and was synthesized for further testing.

### Endoscopy and confocal microscopy revealed target peptide binding *in vivo*

**Figure 2:**
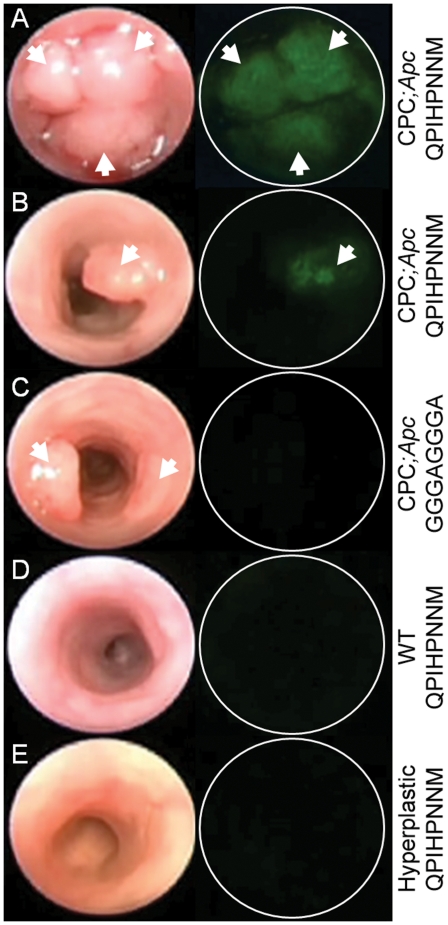
Images from wide-field endoscopy videos after application of fluorescence-labeled peptides. The left and right columns represent frames from white light and fluorescence, respectively. The fluorescent labeled target peptide FITC-Ahx-QPIHPNNM showed positive binding to (A) multiple adenomas and (B) single adenoma in a *CPC;Apc* mouse. (C) The control peptide FITC-Ahx-GGGAGGGA showed minimal binding. The target peptide also showed minimal binding to (D) the lumen of a *CPC;Apc* bred mouse negative for Cre recombinase (control litter mate) and (E) the hyperplastic epithelium in a mutant *Kras* mouse model. White arrows identify adenomas.

**Figure 3:**
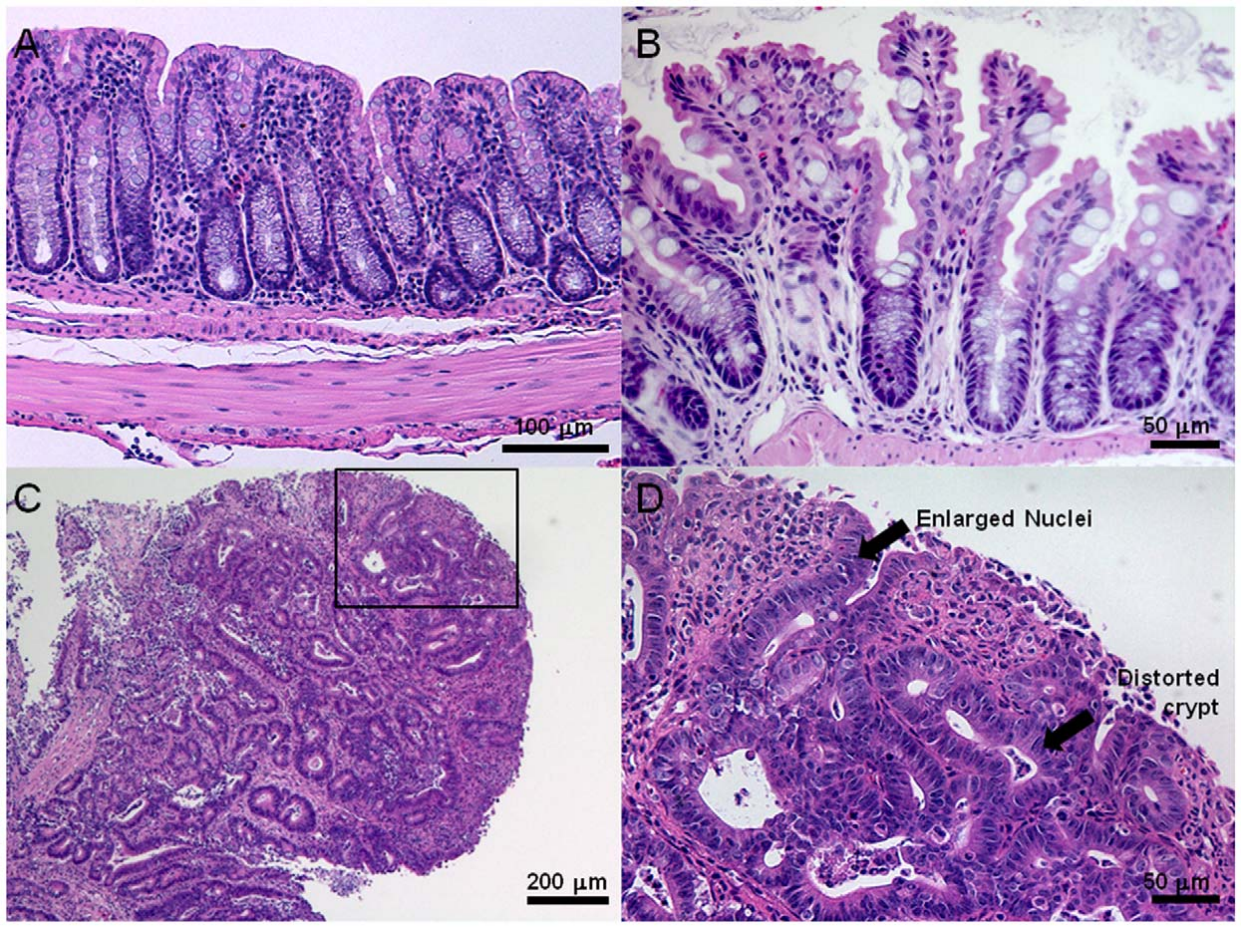
Representative histology from (A) normal colonic mucosa, (B) hyperplastic *Kras*, and (C–D) adenoma in *CPC;Apc* mice. C: An advanced dysplastic adenoma >1 mm in size. D: Higher magnification (scale bar 50 µm) of dysplasia (boxed region of C) displaying enlarged nuclei, hyperchromaticity and distorted crypts.

The *in vivo* endoscopy images revealed that the target peptide (FITC-Ahx-QPIHPNNM) bound to the colonic adenomas and provided greater fluorescence signal when compared to the control peptide (FITC-Ahx-GGGAGGGA), Fig. 2 (Panels A–C). Furthermore, FITC-Ahx-QPIHPNNM showed minimal background binding to adjacent non-adenomatous colon tissue during endoscopy. Also, the FITC-Ahx-QPIHPNNM peptide did not bind to colonic mucosa from control mice lacking the Cre recombinase transgene (Fig. 2D) or to hyperplastic colonic epithelial tissue found in mice with conditional activation of a mutant *Kras* allele in colonic epithelium (Fig. 2E). Representative histology for A) normal (scale bar 100 µm), B) hyperplastic (scale bar 50 µm), and C,D) dysplastic (scale bar 200 and 50 µm, respectively) colonic mucosa are shown in Fig. 3.

Adenomas that developed at approximately 4 cm proximal from the anal verge of the mouse were minimally raised above the mucosal surface, but their morphology, as observed via endoscopy, was not as polypoid as more distal adenomas. Neither the target or control peptide bound to the more proximal and minimally raised lesions. The target peptide may not have bound, because the biopanning experiments were initially performed using visible, polypoid adenomas (i.e., CPC;*Apc* mice of 8 to 9 months of age). Data points for both the target and control peptide on these minimally raised lesions at 4 cm were not used in data analysis, as there were few adenomas of this kind for each peptide tested that were detected.

**Figure 4:**
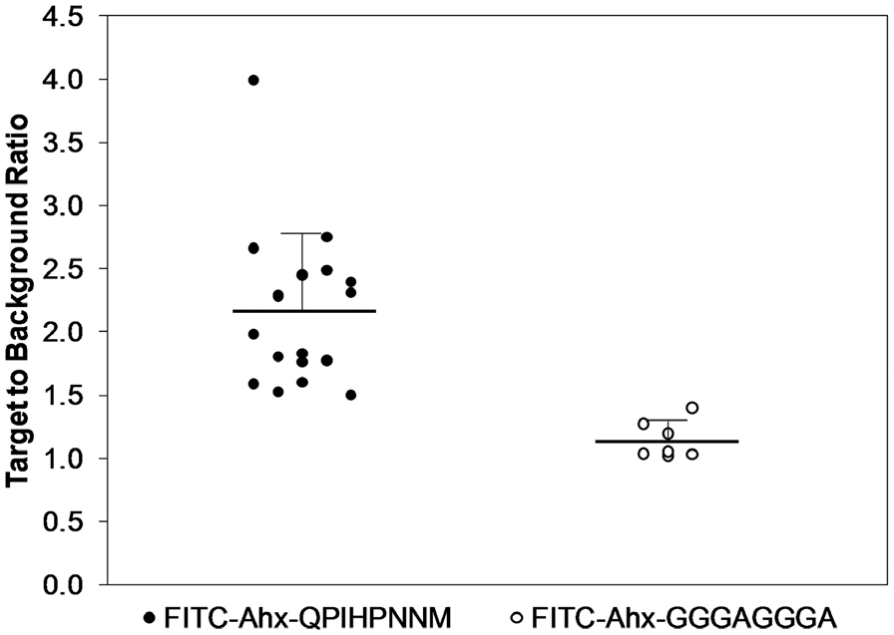
Quantitative analysis of peptide adsorption to distal colonic adenomas showing target peptide FITC-Ahx-QPIHPNNM (2.17±0.61, n = 18) binds greater to the adenomas when compared to the control peptide FITC-Ahx-GGGAGGGA (1.14±0.15, n = 7). The mean of each group is represented by a horizontal black line with one standard deviation from the mean displayed. Non-parametric Mann-Whitney independent samples analysis demonstrates target peptide binds more significantly than control, p<0.01.

Quantitative analysis of peptide adsorption to distal colonic adenomas revealed that the target peptide FITC-Ahx-QPIHPNNM (T/B: 2.17±0.61) binds ∼2-fold greater to the colonic adenomas when compared to the control peptide FITC-Ahx-GGGAGGGA (T/B: 1.14±0.15) (Fig. 4). Non-parametric Mann-Whitney statistical analysis indicated that FITC-Ahx-QPIHPNNM binds in significantly greater amounts to the adenomas than FITC-Ahx-GGGAGGGA, p<0.01. T/B calculations for the FITC-Ahx-QPIHPNNM binding to the control or hyperplastic mice was not possible.

**Figure 5:**
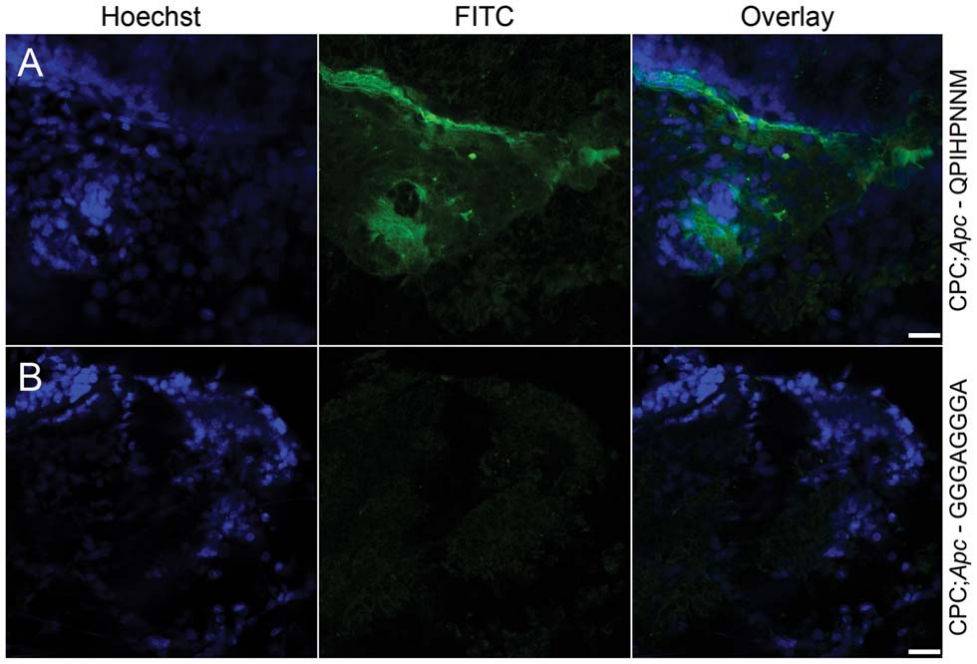
Confocal microscopy images showing binding of fluorescence-labeled peptide to colon adenomas. Biopsies were taken after *in vivo* administration via the endoscope of the FITC-Ahx-QPIHPNNM target peptide (A) or the FITC-Ahx-GGGAGGGA control peptide (B) and subsequently imaged. Scale bar, 25 µm.

Confocal microscopy images of biopsied adenomas collected subsequent to peptide administration showed evidence that the target peptide (Fig. 5A) bound more to adenomas than the control peptide (Fig. 5B).

### QPIHPNNM displayed enhanced binding to whole adenomas *ex vivo*

**Figure 6:**
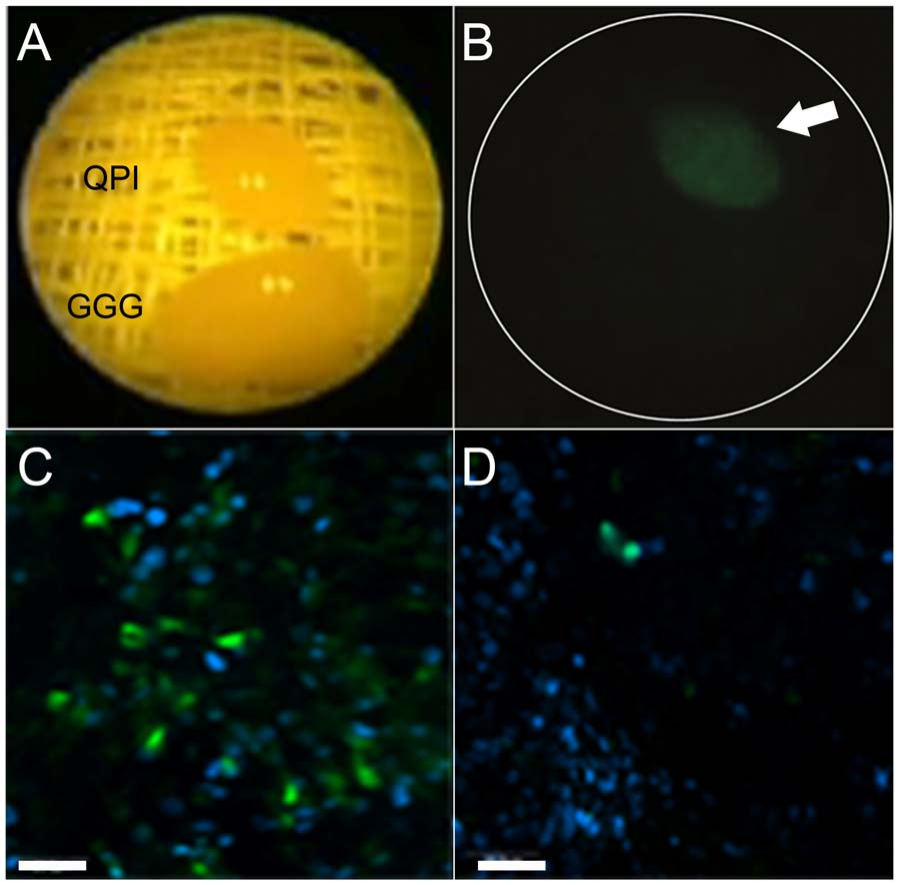
Wide-field and confocal images of excised adenomas incubated with peptide *ex vivo*. (A) Two polyps visualized endoscopically *ex vivo* under conventional white light illumination. (B) *Ex vivo* study shows preferential binding of the fluorescent-labeled target peptide FITC-Ahx-QPIHPNNM compared to the control peptide FITC-Ahx-GGGAGGGA on excised colon. Corresponding representative confocal microscopy images showing increased binding to the adenoma incubated with (C) target peptide when compared to (D) the control. A target to control peptide binding ratio was calculated as 2.51±0.59 (s.d.) for the excised adenoma pairs. Scale bar, 25 µm.

Two adenomas visualized endoscopically *ex vivo* under conventional white light illumination are shown in Fig. 6A. Gross analysis of peptide binding on fluorescence revealed that the FITC-Ahx-QPIHPNNM peptide bound to the excised adenomas more robustly than the FITC-Ahx-GGGAGGGA control peptide (Fig. 6B). Comparison between FITC-Ahx-QPIHPNNM and FITC-Ahx-GGGAGGGA binding showed the target peptide bound 2.51±0.59 times greater than the control peptide for the whole adenoma pairs analyzed.

From the *ex vivo* peptide binding experiment, confocal microscopic imaging revealed that the FITC-Ahx-QPIHPNNM candidate peptide showed increased binding to the dysplastic crypts when compared to the FITC-Ahx-GGGAGGGA control peptide (Fig. 6C and 6D). Minimal to no binding was found for the FITC-Ahx-GGGAGGGA control peptide. These results further validate preferential binding of the target peptide to the adenomas.

### Histology of dysplasia and hyperplasia validated

The normal colonic epithelium from the *CPC;Apc* mice showed well organized crypt morphology (Fig. 3A). The epithelium of the hyperplastic *Kras* mice showed hyperplastic characteristics including a serrated morphology (Fig. 3B). The adenomas from the *CPC;Apc* mice used in phage panning, *in vivo* peptide administration, *ex vivo* peptide administration, and biopsies all showed dysplasia, characterized by enlarged nuclei, hyperchromaticity, and distorted crypts and was validated on histology (Fig. 3C and 3D).

## Discussion

Here, we demonstrate targeted detection of colonic dysplasia *in vivo* using a novel fluorescence-labeled peptide selected with phage display methodologies. These findings highlight an innovative strategy for localizing pre-malignant lesions within the epithelium based on suspected alterations in protein expression rather than on gross architectural abnormalities, and the results suggest that the approach may have potential to enhance the diagnostic specificity of endoscopy in the clinical setting. This work also supports the use of genetically engineered mouse tumor models for longitudinal *in vivo* imaging, allowing for repetitive studies and for each animal to be used as its own control. Validation of the targeted approach with fluorescence endoscopy was achieved using a mouse model that spontaneously develops adenomas in the distal colon. Previously developed *Apc^Min^* mouse models demonstrate polyp growth predominately in the small intestine, an anatomical location that cannot be easily reached with endoscopy. Others have generated mouse models that develop tumors in the distal colon using implanted cancer cells [27] or adenovirus activated mutations [28]. However, these models required surgical intervention to generate polyps, and the ensuing response to injury may have some contributing role in target alteration. Tissue targeting peptides have the advantage of being topically applied where the probe can be delivered in high concentrations to saturate over-expressed dysplastic targets. Our results demonstrate that preferential binding of molecular peptide probes towards dysplastic colonic adenomas can be identified via *in vivo* phage panning.

Phage display panning uses an unbiased approach to select short peptides that bind to over-expressed cell surface targets. Research suggests that the T7 phage system possesses decreased sequence bias compared to the M13 phage systems [25]. In the past, we have successfully utilized phage display to identify peptide binders to high grade dysplasia in human Barrett's esophagus [29] and colonic dysplasia [14] using the commercially available M13 phage system. Because the current experiment involved *in vivo* panning, rather than panning on excised tissue or an established cell line, we chose the T7 system to aid in the reduction of non-specific binding. The T7 system provides greater diversity than the M13 system, is extremely stable, amplifies in a few hours instead of overnight, and can display larger peptides on its protein coat, whereas M13 amplification methods can allow phages with better growth abilities to take over the amplification culture which can decrease diversity with successive rounds of panning, complicating data analysis [30]. Knowing these benefits, the T7 system was built and utilized in our panning procedure.

Our results identified many phage binders to the adenomas; however, the objective of our study was to identify and validate a peptide that could target the spontaneous adenomas in our mouse model *in vivo*. The identified peptide could then be used to localize the pre-malignant lesions on imaging. While a drop in peptide specificity was not expected between rounds 3 and 4 (Fig. 1), this drop could have resulted from the necessity of using separate mice in each of these rounds. While adenomas arising in the *CPC;Apc* mice used here, have the same genetic lesions initiating tumor development (i.e., bi-allelic *Apc* defects in the adenomatous cells), the biological factors contributing to the progressive growth of each polyp likely varies within mice as it does in humans. The target peptide, FITC-Ahx-QPIHPNNM, identified during *in vivo* biopanning was found to bind to colonic adenomas approximately 2-fold greater than a control peptide. This binding was validated in a cohort of six mice, all demonstrating binding *in vivo*, displaying a total of eighteen adenomas. In addition, *ex vivo* validation studies that exhibited a similar 2-fold increase in binding of the target peptide compared to the control peptide. The candidate peptide did not bind to the colon epithelium in the hyperplastic *Kras* mice, illustrating that the peptide is specific to dysplastic colonic mucosa. Adenomatous polyps are thought to be precursors to CRC whereas hyperplastic polyps are not [31], [32], suggesting that the target peptide could be binding to a cell surface target unique to dysplastic and/or cancerous cells. Confocal microscopy verified that the target peptide was indeed binding to the adenoma in comparison with the control peptide in both the biopsy and *ex vivo* experiments. Our initial studies used 5′-FITC as a fluorescent probe, for compatibility with our existing endoscopy instrument; however, the QPIHPNNM peptide can be easily labeled with other dyes in the visible and near infrared (NIR) spectrum, where improved image contrast is expected from reduced tissue autofluorescence. Preliminary experiments testing for QPIHPNNM binding to human surgical specimens of colon adenomas have been performed and are reported in Text S1 and Fig. S1.

Peptides with specific binding properties can be isolated using phage display libraries. To our knowledge, colorectal targeting peptides have been found using *in vitro* phage panning on colon carcinoma cells (CPIEDRPMC on HT29 cells [33], HEWSYLAPYPWF on WiDr cells [34], and VHLGYAT on SW480 cells [35]) and *ex vivo* panning on human colonic tissue (SPTKSNS [36] and VRPMPLQ [14]); however, no colorectal targeting peptides have been identified using methods of *in vivo* phage panning. *In vitro* phage panning on established cell lines remove the targeted cells from their native environment, possibly altering cell behavior. *Ex vivo* panning procedures on excised human tissue have limitations in that they must consider the homogeneity of tissue, the time elapsed after being removed from the patient, and the assumption that all patients over-express the same molecular target. *In vivo* phage panning in a GEM model with genetically identical mice can offer the advantage of isolating peptides that directly accumulate into tumor tissue, binding to either endothelial cells within tumor vasculature [37], epithelial cells through extravasation [38], or extracellular matrix. Work published using the fuse5 phage system show phages accumulate into normal CF-1 mouse intestine within 1 hour post injection [39]. Taking into account that phages are capable of extravasation within an hour and that tumor vasculature is more porous than normal tissue, a stringent time point of 10 minutes was used for *in vivo* circulation within the *CPC;Apc* mouse model to isolate phages that first bind to the target tissue. Our current work supports that phage can extravasate to extracellular matrix and epithelial cells, determining upfront the peptides that would be efficacious systemic tumor-targeting agents.

The previously reported peptide VRPMPLQ is a 7-mer peptide that was isolated using human colon tissue with the intent of direct translation of the peptide to the clinic [14], while the currently reported QPIHPNNM peptide is an 8-mer peptide isolated using the spontaneous mouse model (*CPC;Apc*) in an attempt to further understand the mechanism of CRC. This GEM model allows longitudinal studies to be performed, decreasing the number of mice and cost associated with such experiments. Despite the inability of the QPIHPNNM peptide to detect the minimally raised lesions 4 cm proximal from the anal verge as described in the results, the QPIHPNNM may be utilized to detect dysplastic tissue from hyperplastic tissue *in vivo*. Furthermore, the newly discovered peptide, QPIHPNNM, now provides the opportunity to improve our optical probes and instruments to optimize wide-field *in vivo* lesion detection that can eventually be translated to the clinic. We have demonstrated the first wide-field fluorescence binding of a peptide *in vivo*, showing the advancement and feasibility of targeted detection of diseased tissue based on tissue biology rather than anatomical changes *in vivo*. The technique of *in vivo* phage display can also be used to develop additional peptides to test novel methods of multiple delivery or simultaneous detection of peptides using this mouse model.

The confocal microscopy results show that the target peptide specifically binds to dysplastic cells compared to the control peptide; however, the pattern of target peptide binding suggests either cell surface or extracellular matrix binding. The QPIHPNNM molecular target is not known, and the sequence does not show full homology to any known receptor ligands. The QPIHPNNM peptide does have partial homology to the Ep300 protein (QP**PNNM) and an undefined cell adhesion molecule-related/down-regulated by oncogenes precursor (transmembrane protein, QPIHP), with the latter suspected to be involved in the development of colorectal tumors [40], [41].

Various attempts to design control peptides for target peptide validation reported include amino acid replacement [42], [43], incorporation of an unrelated peptide sequenced during panning [44], and peptide scrambling. During the four rounds of T7 phage library panning in the reported experiment, unrelated peptides having the same length (8-mer) as the target peptide identified were not found. Peptide scrambling or amino acid replacement are most commonly implemented if the binding properties of individual amino acids is known. Since no structural and binding site information for the target QPIHPNNM peptide is known, efforts for designing a scrambled peptide or peptide with altered amino acids were not attempted. In short peptides (<10 mer), it has been reported that only few amino acids (2–3) have a significant role in binding, and these kinds of short peptides can show some binding even after being scrambled [45]. The nature of the peptide (i.e., net charge, hydrophobicity, hydrophilicity) can also determine the fate of the binding. Removing functional groups and neutralizing charge on a control peptide have been previously used to demonstrate specific binding of the target peptide [46], [47]. Taking this information into account, we designed our control peptide (GGGAGGGA) to contain the same 5′-FITC fluorophore, Ahx linker, and number of amino acids compared to the target peptide.

To the best of our knowledge, this is the first study to use a mouse model that mimics the progression of human colon cancer to demonstrate the use of fluorescence-labeled peptides to identify and localize dysplasia in wide-field endoscopy. This approach can be generalized to other mouse models for studying cancer development in organs accessible though microendoscopic instruments and ultimately to clinical detection and localization in human disease.

## Materials and Methods

### T7 Library construction

The T7 library was constructed with the T7Select 10-3b vector as reported in the T7Select System Manual (Novagen, Gibbstown, NJ). Briefly, random oligonucleotide insert DNA for the X_18_ library was synthesized as follows: 5′-AAC TGC AAG CTT TTA-(MNN)_18_-ACC ACC ACC AGA ATT CGG ATC CCC GAG CAT-3′ (where N represents an equi-molar ratio of each nucleotide and M is an equi-molar ratio of adenine and cytosine). The amino acid translation of the complementary nucleotide sequence is: MLGDPNSGGGX_18_. The insert DNA was incubated with a complementary extension primer (5′-ATG CTC GGG GAT CCG AAT TCTGGT-3′), Klenow enzyme (New England Biolabs, Beverly, MA), and deoxyribonucleotide triphosphates (Novagen) to form the complementary DNA strand. This was digested with *Eco*R1 and *Hind*III restriction endonucleases (New England Biolabs). Following phenol/chloroform extraction and ethanol precipitation, the purified fragments were ligated into predigested T7Select 10-3b vector by T4 DNA ligase (Novagen). The ligation reaction was incubated at 16°C, subjected to *in vitro* packaging, and titered to determine the number of plaque forming units (pfu). The remaining solution was amplified in isopropyl β-D-1-thiogalactopyranoside (IPTG)-induced BLT5615 until lysis. The lysate was titered and stored at −80°C in glycerol.

### Mouse models

Mice were cared for under the approval of the University Committee on the Use and Care of Animals, University of Michigan (UCUCA, Approved protocol 09881). The genetically engineered mouse model (*CPC;Apc*) that produce adenomatous polyps are mice containing Cre recombinase under the control of the Cdx2 promoter (*CDX2P-9.5NLS-Cre*) and a floxed allele of the *APC* gene [12]. This somatic mutation in an *Apc* allele, which leads to a truncated *Apc* protein, causes the development of adenomas in the distal colon as early as 10 weeks. Mice exhibiting hyperplastic colon epithelium (hyperplastic *Kras*) were generated by breeding mice with mutant *Kras* allele whose transcription is prevented by a stop element flanked by LoxP recombination sites (*Kras^LSL-G12D/+^*) [48], [49] with transgenic mice expressing Cre in the epithelial cells of a significant fraction of crypts of the terminal ileum, cecum and colon (*CDX2P9.5-G22Cre*) [50]. All mice were housed in specific pathogen-free conditions and supplied water *ad libitum* throughout the study.

### *In vivo* phage panning procedure

A 9 month old *CPC;Apc* mouse was injected via tail vein with 1×10^11^ pfu of the parent T7-18mer library. The library was allowed to circulate for 10 min, after which the mouse was euthanized and organs were harvested and kept on ice. The bound phages were recovered by homogenizing each tissue or organ (colon adenomas, normal colonic mucosa) (Bio-gen Pro 200) in DMEM-PI (Dulbecco's Modified Eagle Medium plus protease inhibitors: 1 mM phenylmethanesulfonylfluoride (PMSF), 20 µg/mL aprotinin, and 1 µg/mL leupeptin) [36], [37]. The tissue samples were washed 3× with ice-cold washing medium (DMEM-PI containing 1% bovine serum albumin), centrifuging for 5 min at 3000 rpm between each wash. After the last wash, freshly starved bacteria were added to each tissue homogenate and incubated for 30 min at room temperature (RT). Pre-warmed Luria-Bertani (LB) medium with carbenicillin (50 µg/mL) was then added to the bacteria-homogenate solution and incubated another 30 min at RT. The supernatant was recovered after centrifugation and titered to determine the number of bound phage within each tissue tested. This procedure constituted one round of panning. After two rounds of panning, the recovered phages that bound to the colon adenomas were cleared twice: once against a homogenized tissue cocktail consisting of colon, kidney, liver and heart and a second time against normal murine colon. A total of four rounds of phage panning were performed, with amplification of the recovered eluate after each panning round. The input phage number (1×10^11^ pfu) was kept constant for each round of panning. All T7 DNA was sequenced per Novagen's suggested protocol using a DNA sequencer (Applied Biosystems, 3730XL DNA Analyzer, UM DNA Core). The number of phages bound to each organ or tissue was calculated as the output pfu/(input pfu×tissue mass).

### Peptide synthesis

The peptides were synthesized using standard Fmoc-chemistry by solid phase synthesis [51]. To ensure the phage and synthetic peptide were expressed with the same orientation, the candidate peptide was synthesized with 5′-fluorescein isothiocyanate (FITC) attached to the amino terminus of the peptide via amino hexanoic acid linker (Ahx). Deprotection and cleavage of the peptides were achieved by treatment with a cleavage cocktail of trifluoroacetic acid (TFA)/Tri-isopropylsilane/water (9.5/0.25/0.25, v/v/v) at RT for 3–4 hours. After cleavage of the product from the resin, the peptides were purified by preparative-HPLC using a water (0.1% TFA)-acetonitrile (0.1%TFA) gradient (2–33% acetonitrile over 31 min) (Waters Breeze HPLC, Milford, MA). The peptides were characterized by an ESI mass spectrometer (Micromass LCT Time-of-Flight mass spectrometer with Electrospray, target peptide (FITC-Ahx-QPIHPNNM) mass (Calc. 1451.89, Obs. 1451.7 [M+H]^+^)). The purity (>95%) of the compound was confirmed by analytical HPLC on a C_18_ column. As the side chains of amino acids can have significant roles in binding with their targets, a control peptide containing Gly and Ala (GGGAGGGA), which does not have any functional groups on the amino acid side chains, was synthesized using same methods (control peptide (FITC-Ahx-GGGAGGGA) mass (Calc. 1004.08 Obs. 1004.4 [M+H]^+^)). All peptides were reconstituted in 1× phosphate buffered saline (PBS) at 500 µM and further diluted in 1× PBS as necessary.

### Small animal endoscopy and peptide administration

Prior to peptide administration, the colon was prepped using a tap water lavage. Using a small animal endoscope (Karl Storz Veterinary Endoscopy, Goleta, CA) with a 3Fr instrument channel for performing biopsy, adenomas suitable for peptide administration were located, and the colon was rinsed with water until all mucous was removed [52]. An adenoma was determined not suitable for peptide administration if the adenoma was large enough (approximately >4 mm) to make navigation with the animal endoscope difficult, as peptide delivery and washing would be inconsistent. Adenomas from approximately 0.5–4 mm that were not covered in debris, including stool and mucous, were included in the study.

The fluorescence-labeled peptide was delivered at a concentration of 100 µM in 1× PBS through an instrument channel. During peptide administration, approximately 1 mL of peptide was delivered to the lower 4 cm of colon in each mouse tested. The peptide was allowed to incubate for 5 min after which the colon was cleansed 3× with a tap water to remove the unbound peptide. Prior to imaging, the colon was inspected for residual peptide solution, and when clean, the colon was insufflated with air and imaged. Fluorescence excitation in the spectral regime from 450 to 475 nm was produced with a bandpass filter that can be manually switched to the optical path of a 175 W Nova Xenon light source and was delivered to the endoscope via a 3 mm diameter fluid light cable (250 cm length). Fluorescence images were collected using a 510 nm long-pass filter to block the excitation light and was detected with a 3-chip color camera with an integrated parfocal zoom lens. Real time video was recorded via firewire connected to a pc. Adenomas in *CPC;Apc* mice developing distal colonic adenomas (QPIHPNNM n = 6 mice having n = 18 adenomas; GGGAGGGA n = 4 mice having n = 7 adenomas) in addition to Cre recombinase negative littermate controls (QPIHPNNM n = 2 mice) and hyperplastic *Kras* mice (QPIHPNNM n = 3 mice) were imaged with both white light and fluorescence endoscopy. The *CPC;Apc* mice imaged using fluorescence endoscopy ranged in age from 3 to 5 months.

### Fluorescent image analysis

Videos collected during endoscopy were exported as .avi video files and converted into sequential .png images using Apple QuickTime. Consecutive white light and fluorescence images for each adenoma analyzed were imported into NIH Image J. The white light image was utilized to draw a region of interest (ROI) around the adenoma or adjacent normal appearing colonic mucosa which was then superimposed onto the fluorescent image (Fig. S2). Mean values were calculated for each ROI, and an adenoma/normal colon, or a target to background ratio (T/B), value was then calculated for each adenoma. Target to background ratios were not calculated for Cre recombinase negative littermate control mice or hyperplastic *Kras* mice, because these mice were devoid of adenomas.

### *Ex vivo* Peptide Binding

Adenomas from *CPC;Apc* mice (n = 3 mice, 5 adenoma pairs) were excised and washed 3× in PBS. The adenomas were incubated in 100 µM peptide solution for 5 min and subsequently washed 3× with 1× PBS. Adenomas were grossly imaged using the small animal endoscope to view binding to the adenoma as a whole. Pairs of adenomas (one candidate, one control) were imaged at a time to allow for the calculation of a target peptide to control peptide binding ratio for each pair analyzed. An average binding ratio was calculated ± one standard deviation. A similar procedure to determine mean values for image analysis was followed for the *ex vivo* adenomas as described above for the *in vivo* adenomas.

### Confocal Fluorescence Imaging

Preferential binding of the target peptide compared to the control peptide on adenomatous polyps was confirmed with confocal microscopy after both *in vivo* and *ex vivo* testing. From the *in vivo* experiment, a biopsy was taken after 5′-FITC-labeled peptide administration *in vivo*, incubated in 1 µg/mL Hoechst dye for 5 min to stain living nuclei, rinsed 3× with 1× PBS and imaged on a Leica TCS SP5 Microscope (Leica Microsystems, Bannockburn, IL). From the *ex vivo* experiment, excised adenomas incubated with peptide *ex vivo* were subsequently incubated in 1 µg/mL Hoechst dye for 5 min to stain living nuclei, rinsed 3× with 1× PBS and imaged on an Olympus FluoView 500 laser scanning confocal microscope (Olympus Corp, Tokyo, Japan).

### Statistics

All results are shown as mean ± one standard deviation. A non-parametric Mann-Whitney Independent Samples test was performed to determine statistical significance which was defined as p<0.05 between target and control peptide binding to colonic adenomas (PASW Statistics 18, Chicago, IL).

### Histology

All tissue was fixed in phosphate-buffered formalin for 24 hours, paraffin-embedded and sectioned into 10 µm thin slices and stained with hematoxylin and eosin (H&E). Histological images were captured using an Axioskop2 upright microscope (Carl Zeiss Microimaging, Inc. Thornwood, NY).

## Supporting Information

Figure S1 **Preliminary fluorescent peptide binding to human surgical specimens of non-neoplastic or neoplastic human colon tissue showing QPIHPNNM binds to dysplastic adenoma but not to normal tissue.** The GGGAGGGA control peptide displayed minimal binding to normal colon tissue from the same patient. Scale bar 20 µm.
(TIF)

Figure S2 **The region of interest (ROI) for each polyp was selected using a series of image frames exported upon conversion from .avi video file to serial .png images using Apple QuickTime Player.** The Storz small animal endoscope has an excitation filter wheel that can be rotated from the fluorescence excitation filter of 450–475 nm to no excitation filter which produces an image similar to the wide-field white light view when the filters are removed from the endoscope. The image produced when no excitation filter is selected (referred to as “filtered white light”) is still subject to the emission filter of 510–700 nm that is positioned in front of the camera, and is, therefore, dimmer than the white light images shown in Fig. 2. Using the filtered white light image (Fig. S2A), a region of interest around the entire adenoma (yellow polygon) was drawn using the polygon selection tool in the NIH ImageJ software. This ROI was then superimposed onto the fluorescence image (Fig. S2B) taken within ten frames of video. The example shown in Fig. S2 uses images (A) filtered white light and (B) fluorescence as the exported .png frames. The ROI for the adjacent suspected normal colon tissue (white polygon) was chosen using the same method.
(TIF)

Text S1 **Preliminary experiments testing for QPIHPNNM binding to human surgical specimens of colon adenomas have been performed.**
(DOCX)
