# Supplementary figures and images for: In Vivo Fluorescence-Based Endoscopic Detection of Colon Dysplasia in the Mouse Using a Novel Peptide Probe
Source: PLoS One. 2011 Mar 8;6(3):e17384. doi: 10.1371/journal.pone.0017384 (PMC3050896; doi:10.1371/journal.pone.0017384)

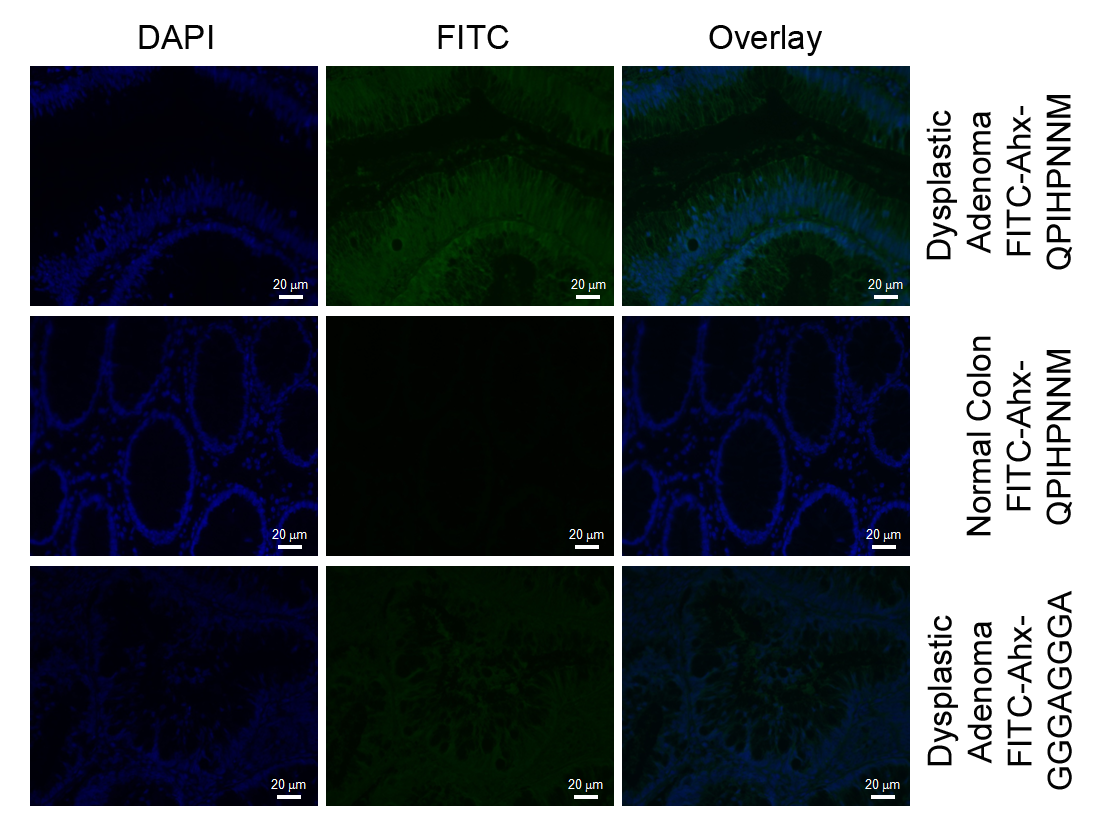

Supplement: Figure S1 — Preliminary fluorescent peptide binding to human surgical specimens of non-neoplastic or neoplastic human colon tissue showing QPIHPNNM binds to dysplastic adenoma but not to normal tissue. The GGGAGGGA control peptide displayed minimal binding to normal colon tissue from the same patient. Scale bar 20 µm. (TIF) [file pone.0017384.s001.tif]

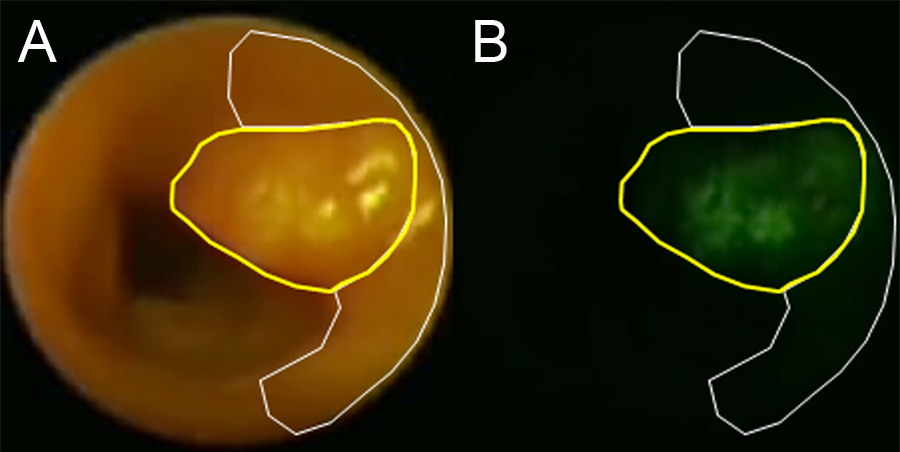

Supplement: Figure S2 — The region of interest (ROI) for each polyp was selected using a series of image frames exported upon conversion from .avi video file to serial .png images using Apple QuickTime Player. The Storz small animal endoscope has an excitation filter wheel that can be rotated from the fluorescence excitation filter of 450–475 nm to no excitation filter which produces an image similar to the wide-field white light view when the filters are removed from the endoscope. The image produced when no excitation filter is selected (referred to as “filtered white light”) is still subject to the emission filter of 510–700 nm that is positioned in front of the camera, and is, therefore, dimmer than the white light images shown in Fig. 2. Using the filtered white light image (Fig. S2A), a region of interest around the entire adenoma (yellow polygon) was drawn using the polygon selection tool in the NIH ImageJ software. This ROI was then superimposed onto the fluorescence image (Fig. S2B) taken within ten frames of video. The example shown in Fig. S2 uses images (A) filtered white light and (B) fluorescence as the exported .png frames. The ROI for the adjacent suspected normal colon tissue (white polygon) was chosen using the same method. (TIF) [file pone.0017384.s002.tif]
