# Supplementary material for: In Vivo Fluorescence-Based Endoscopic Detection of Colon Dysplasia in the Mouse Using a Novel Peptide Probe
Source: PLoS One. 2011 Mar 8;6(3):e17384. doi: 10.1371/journal.pone.0017384 (PMC3050896; doi:10.1371/journal.pone.0017384)
Supplement: Text S1 — Preliminary experiments testing for QPIHPNNM binding to human surgical specimens of colon adenomas have been performed. (DOCX) [file pone.0017384.s003.docx]

**Supplementary Text**

Preliminary experiments testing for QPIHPNNM binding to human surgical specimens of colon adenomas have been performed. In brief, frozen 10 µm thick sections non-neoplastic or neoplastic human colon tissue were washed with PBS and incubated with FITC labeled peptide (10 µM) for 15 minutes at room temperature. After three 1 minute washes in PBS, sections were fixed in 4% paraformaldehyde for 10 minutes, washed again with PBS, mounted with Prolong Gold reagent with DAPI, and imaged under a fluorescence microscope (Zeiss Axioskop2). Our preliminary data (Fig. S1) show only QPIHPNNM target peptide binding to adenomatous tissue. The target peptide displayed minimal binding to normal colon tissue from the same patient.
